# Supplementary material for: Root tip excision‐induced exodermis lignification impacts lateral root emergence in Brachypodium distachyon
Source: New Phytol. 2026 Jan 2;249(6):2894–908. doi: 10.1111/nph.70883 (PMC12917469; doi:10.1111/nph.70883)
Supplement: Supplementary file 1 — Fig. S1 Bd21 and Bd21‐3 display distinct lateral root emergence dynamics following root tip excision. Fig. S2 Lateral root primordium development is synchronized and comparable between accessions. Fig. S3 Bd21 ‘pine‐tree’ and Bd21‐3 ‘fishbone’ root phenotypes 60 h after root tip excision. Fig. S4 Orthogroups with multiple copies in the Bd21 accession are enriched for stress and DNA repair functions. Fig. S5 RNA‐seq analysis highlights distinct early and late transcriptional responses in both B. distachyon accessions. Fig. S6 Number of responsive genes to mechanical stimulation (touch) and root tip excision in B. distachyon roots. Fig. S7 Molecular function gene ontology enrichment of deregulated genes in both Bd21 and Bd21‐3 in response to root tip excision. Fig. S8 Gene Ontology enrichment analysis for differentially expressed genes. Fig. S9 Comparative transcriptomic analysis reveals a divergent cell wall remodelling response in Bd21 and Bd21‐3 after root tip excision. Fig. S10 Spatio‐temporal analysis of lignin deposition after root tip excision. Fig. S11 Increased lignin deposition following root tip excision in Bd21‐3. Fig. S12 Lignin monomer composition is elevated in the upper root zone of Bd21‐3. Fig. S13 Expression dynamics of selected MYB transcription factor homologs following root tip excision. Fig. S14 Bd21 and Bd21‐3 show similar exodermis lignification patterns along the root tip–base axis. Fig. S15 Accession‐specific patterns of exodermis lignification during lateral root emergence. Fig. S16 Reduced exodermal lignin accumulation in Brachypodium roots following piperonylic acid (PA) treatment. [file NPH-249-2894-s001.pdf]

New Phytologist Supporting Information

Article title : Root tip excision-induced exodermis lignification impacts lateral root emergence in *Brachypodium distachyon*

Authors : Kevin Bellande, Cristovão De Jesus Vieira Teixeira, Marius Malai, Angelina D'Orlando, Léa Perez, Richard Sibout, Anne C. Roulin, Joop E.M. Vermeer and Thomas Badet

Article acceptance date: 11 December 2025

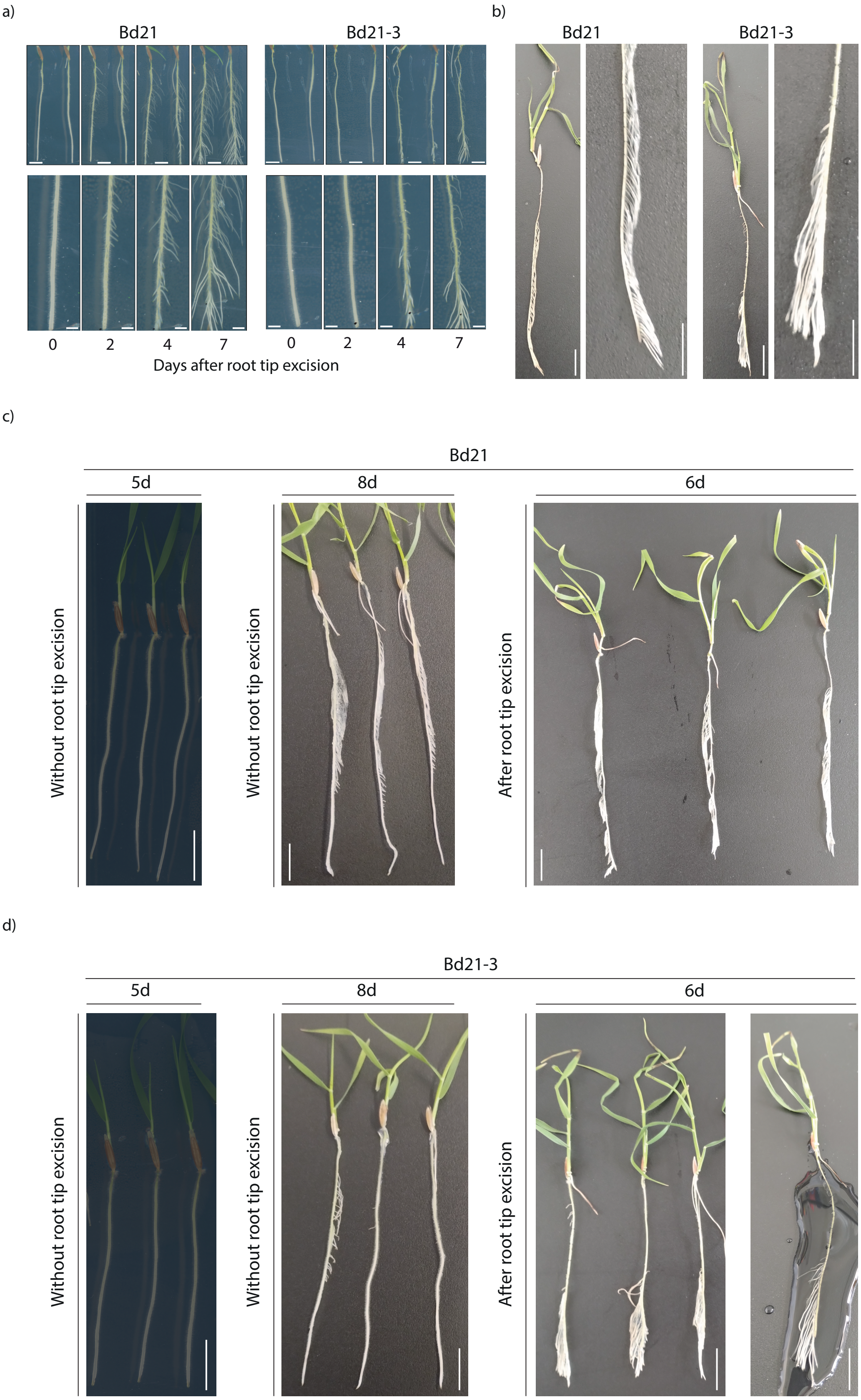

Figure S1. Bd21 and Bd21-3 display distinct lateral root emergence dynamics following root tip excision. a) Time-course of lateral root emergence dynamics after

root tip excision. Representative images of whole root systems of Bd21 and Bd21-3 at 0, 2, 4, and 7 days after root tip excision. The lower panels provide magnified views showing the emergence of lateral roots along the entire root in Bd21 and the restricted emergence in the upper regions of Bd21-3. Scale bars: Upper panel, 5 mm; lower panel, 3 mm. (b) Magnified comparison of whole root systems between Bd21 and Bd21-3, illustrating the difference in lateral root density and distribution 6 days after root tip excision. (c) Root system architecture in Bd21 seedlings at 5 days (5d) and 8 days (8d) without root tip excision (untreated control) and at 6 days (6d) after root tip excision. (d) Root system architecture in Bd21-3 seedlings at 5 days (5d) and 8 days (8d) without root tip excision (untreated control) and at 6 days (6d) after root tip excision, highlighting the difference in emergence patterns compared to Bd21. Scale bars: 1 cm.

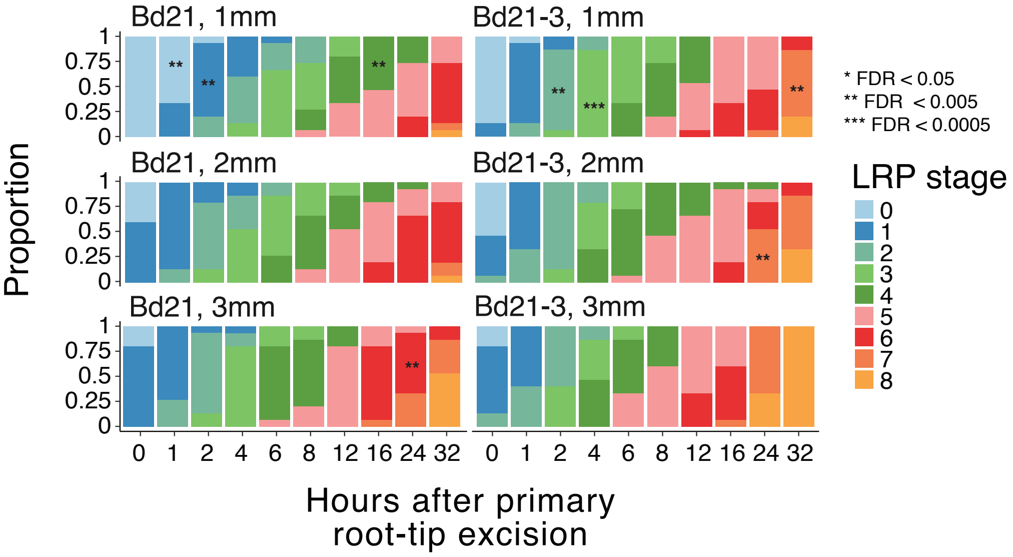

Figure S2. Lateral root primordium development is synchronized and comparable between accessions. Quantification of lateral root primordium stages within the first 1, 2, and 3 mm of the root above the excision site over a 32-hour time course. The stacked bar charts show the proportion of lateral root primordia at each developmental stage (0-8) for Bd21 and Bd21-3. Stars indicate statistical significance based on Fisher's exact test: *FDR < 0.05, **FDR < 0.005, ***FDR < 0.0005.

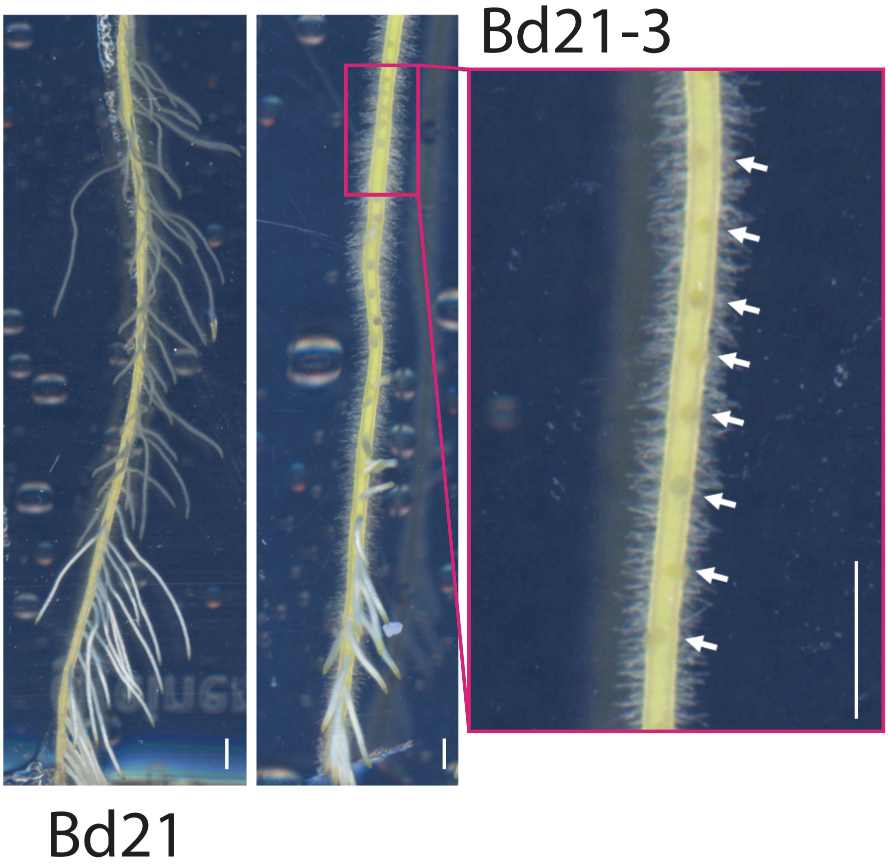

Figure S3. Bd21 “pine-tree” and Bd21-3 “fishbone” root phenotypes 60h after root tip excision. Magnified view of non-emerged lateral root primordia in Bd21-3. Representative images of Bd21 and Bd21-3 roots at 60 hours after root tip excision. Scale bars: 2.5 mm.

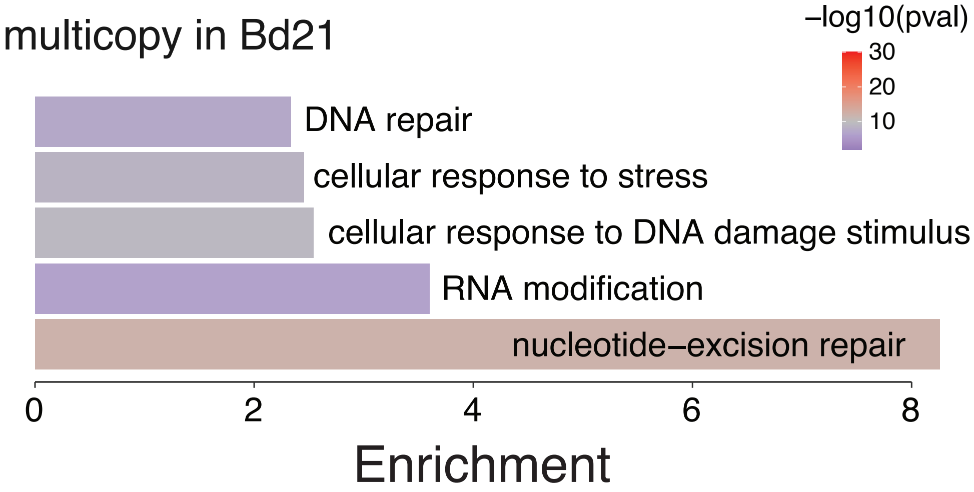

Figure S4. Orthogroups with multiple copies in the Bd21 accession are enriched for stress and DNA repair functions. GO enrichment analysis for biological processes associated with genes found in multicopy orthogroups specific to the Bd21 accession. The bar plot shows the enrichment scores for the most significantly overrepresented GO terms, which are predominantly related to DNA repair and cellular responses to stress. The colour of each bar corresponds to the statistical significance ($-\log_{10}$(p-value)).

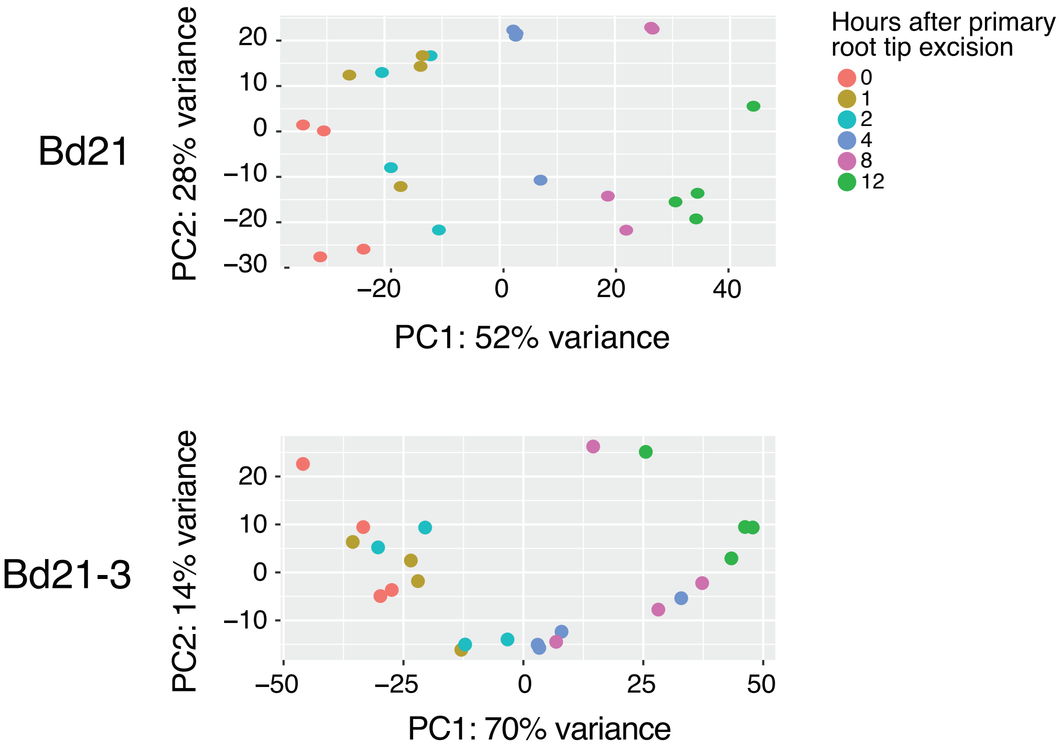

Figure S5. RNA-seq analysis highlights distinct early and late transcriptional responses in both *B. distachyon* accessions. Principal component analysis (PCA) of the transcriptomic data from root tissues collected at 0, 1, 2, 4, 8, and 12 hours post root tip excision. PCA were computed after variance stabilizing transformation on a subsample of the top 500 features by variance across all conditions.

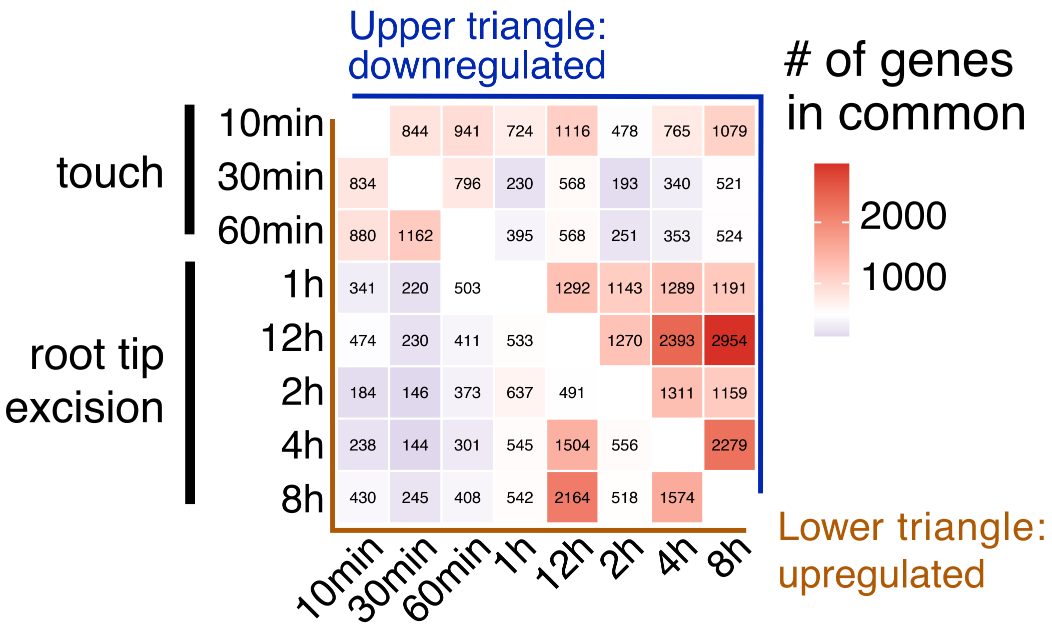

Figure S6. Number of responsive genes to mechanical stimulation (touch) and root tip excision in *B. distachyon* roots (Coomey *et al*., 2024). Heatmap of pairwise comparisons depicting the number of shared differentially expressed genes. The upper triangle (above the diagonal) represents the number of genes that are commonly downregulated compared to the basal state (time 0). The lower triangle (below the diagonal) represents the number of genes that are commonly upregulated compared to the basal state (time 0). Conditions 10, 30 and 60 minutes are from the root touch stimulations study by (Coomey *et al*, 2024) while 1, 2, 4, 8 and 12 hours are from the present study.

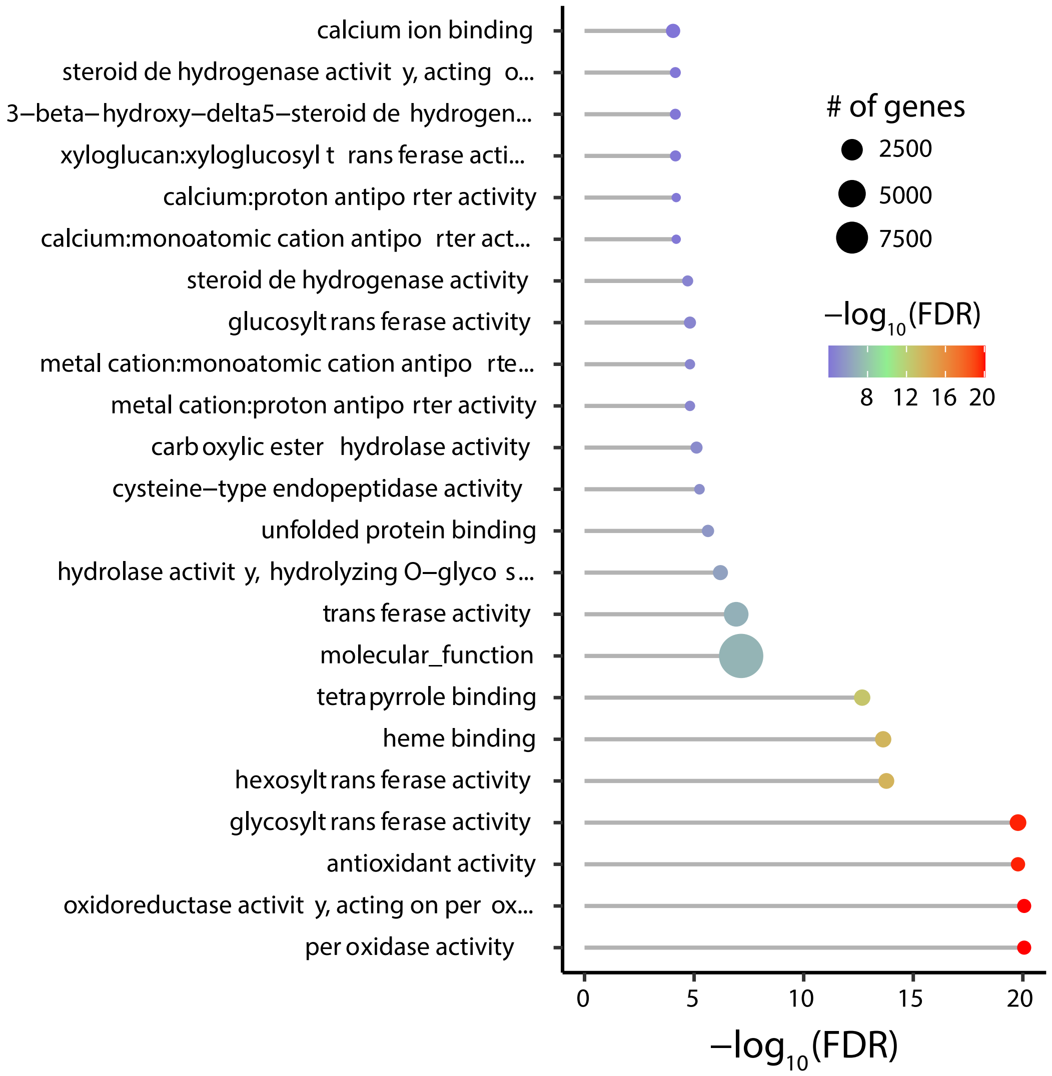

Figure S7. Molecular function gene ontology enrichment of de-regulated genes in both Bd21 and Bd21-3 in response to root tip excision. Molecular Function Gene Ontology (GO) enrichment of the differentially expressed genes (DEGs) identified in the comparative transcriptomic analysis following root tip excision in both *B. distachyon* accessions, Bd21 and Bd21-3. P-values were adjusted according to the Benjamini & Hochberg method (FDR) and results are displayed for functions below the 0.0001 threshold. Dot size represents the number of genes falling into the category.

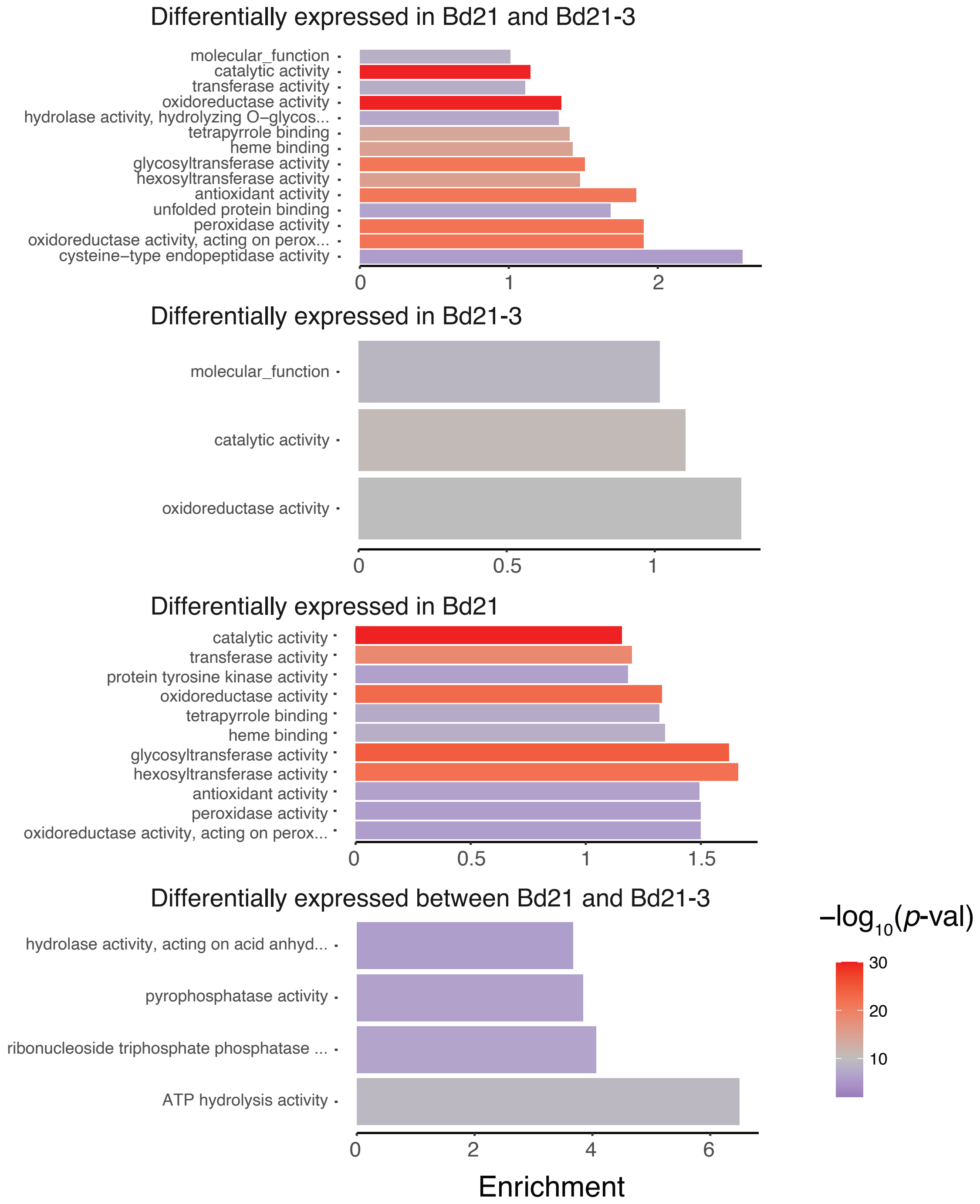

Figure S8. Gene Ontology enrichment analysis for differentially expressed genes. The bar charts display the GO enrichment analysis for different subsets of differentially expressed genes following root tip excision, focusing on the "molecular function" category. The x-axis represents the enrichment score, and the colour of the bars indicates the statistical significance (-log10(p-value)). (Top panel) GO terms enriched for genes that are differentially expressed in both *Bd21* and *Bd21-3*. (Second

panel) GO terms enriched for genes differentially expressed only in *Bd21-3*. (Third panel) GO terms enriched for genes differentially expressed only in *Bd21*. (Bottom panel) GO terms enriched for the 240 genes that show opposite regulation between the two accessions.

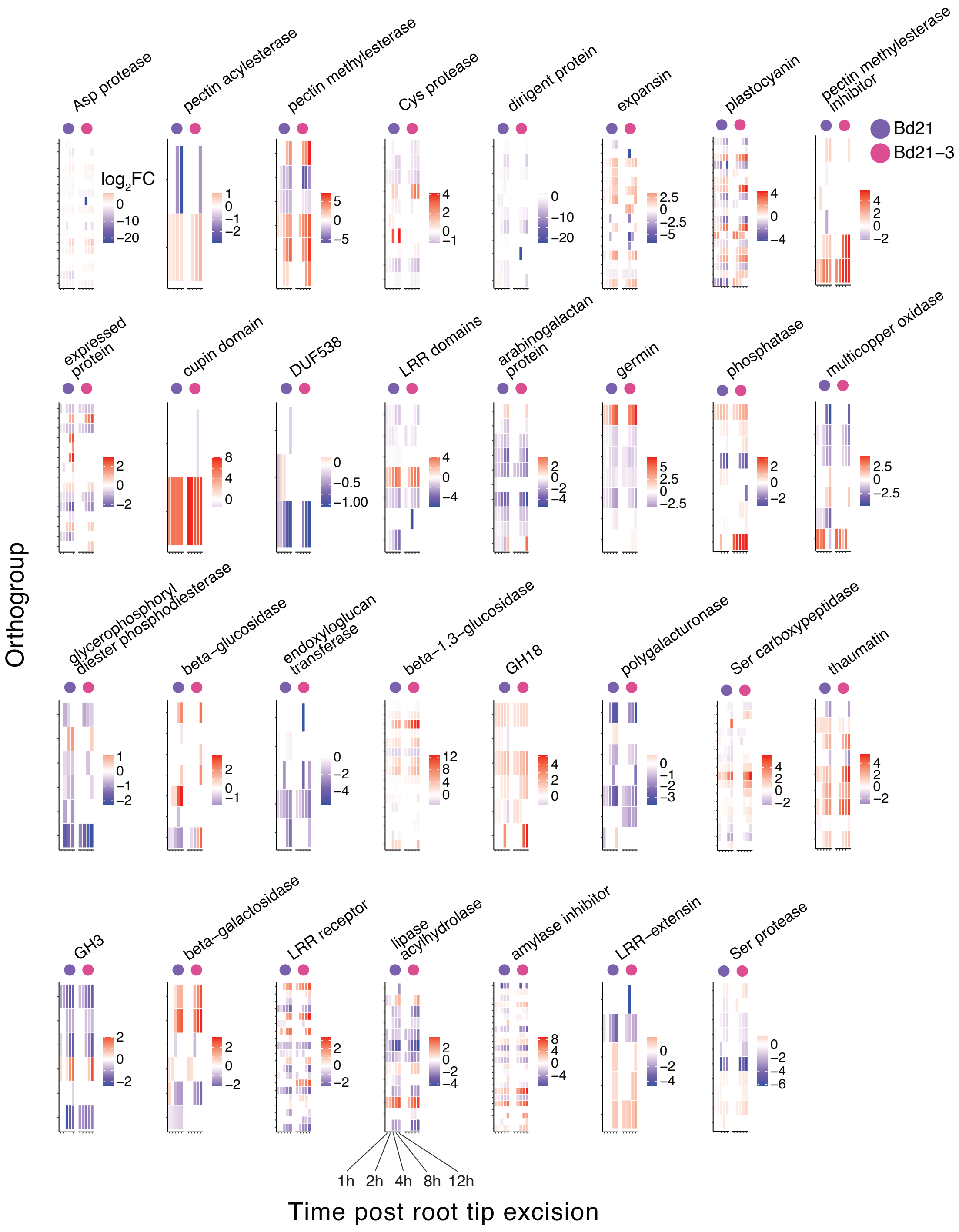

Figure S9. Comparative transcriptomic analysis reveals a divergent cell wall

remodelling response in Bd21 and Bd21-3 after root tip excision. Heatmaps showing the expression dynamics ($\log_2$ fold-change) for individual genes within selected cell wall-related enzyme families. Each row represents a single gene, and its expression is shown over the 12-hour time course for both Bd21 and Bd21-3.

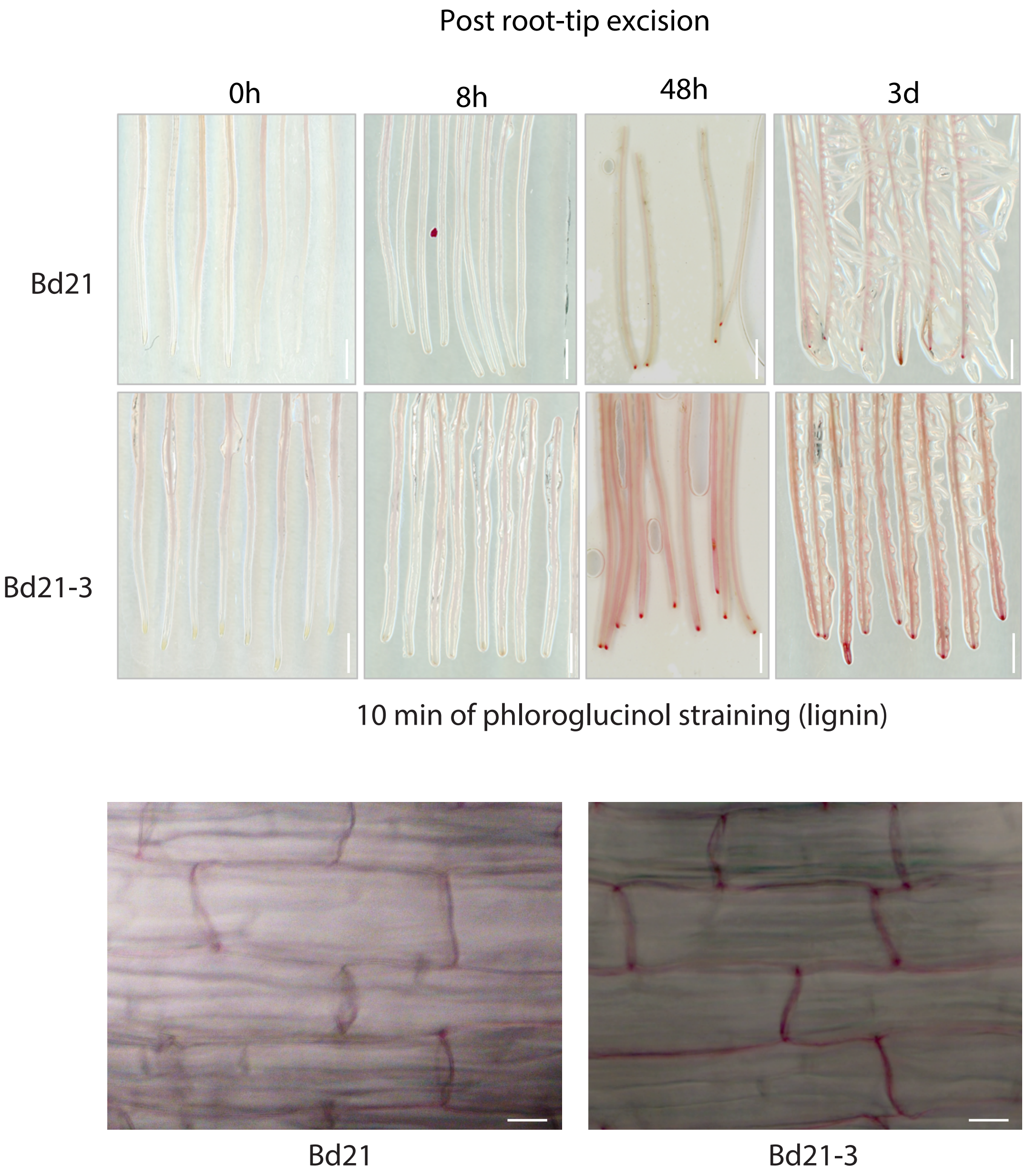

Figure S10. Spatio-temporal analysis of lignin deposition after root tip excision. This figure visualizes the dynamics of lignin deposition using phloroglucinol staining. The top panel shows a time course at 0, 8, 40 hours, and 3 days after root tip excision in the roots of Bd21 and Bd21-3. The bottom panel provides magnified images of the root epidermis, highlighting the strong and distinct lignification of cell walls in Bd21-3 compared to the faint staining in Bd21. Scale bars, upper panel: 5 mm; lower panel: 10 μm.

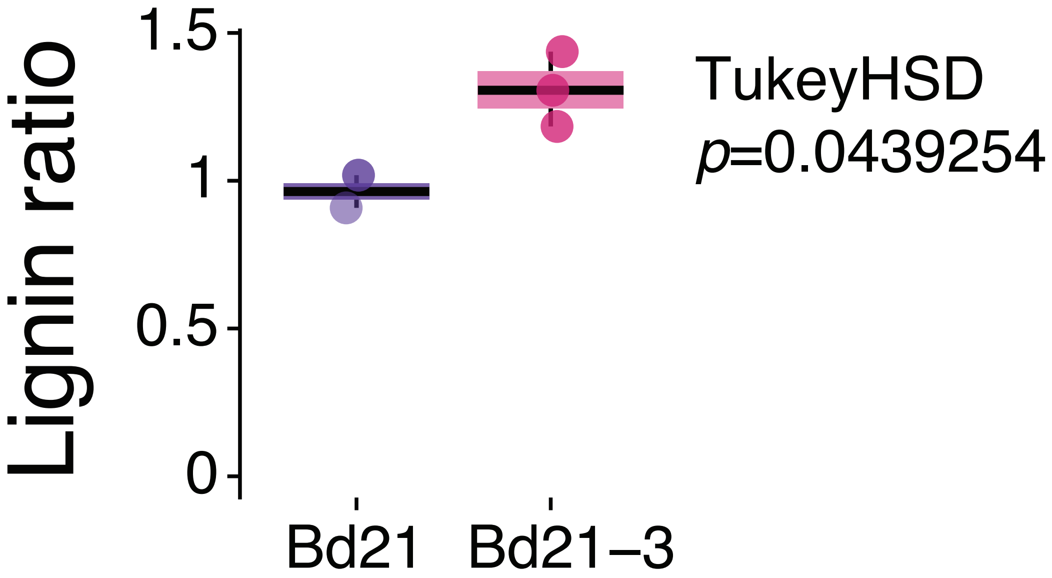

Figure S11. Increased lignin deposition following root tip excision in Bd21-3. Quantification of total lignin content (%) in whole roots of Bd21 and Bd21-3 as determined by the CASA method 0h and 40h after root tip excision (see Fig. 3b). Values are the ratio of lignin content compared to time 0. Each point represents a biological replicate.

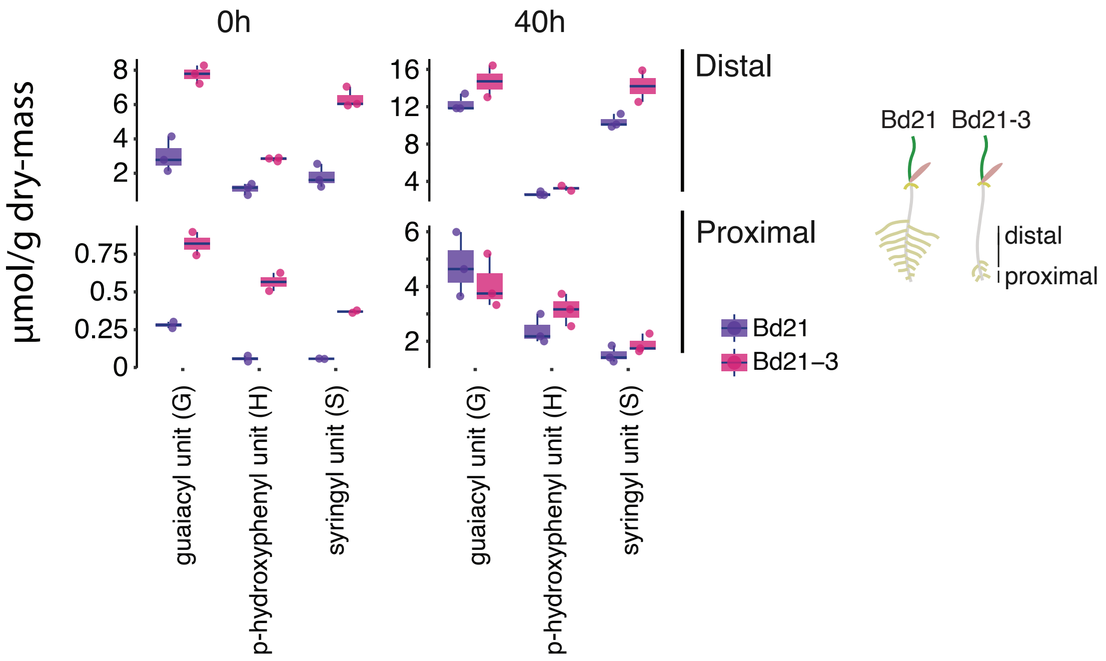

Figure S12. **Lignin monomer composition is elevated in the upper root zone of Bd21-3.** Spatially resolved quantification of lignin monolignol composition in proximal and upper root regions at 0- and 40-hours post excision. The box plots show the amounts (µmol/g) of guaiacyl (G), p-hydroxyphenyl (H), and syringyl (S) units.

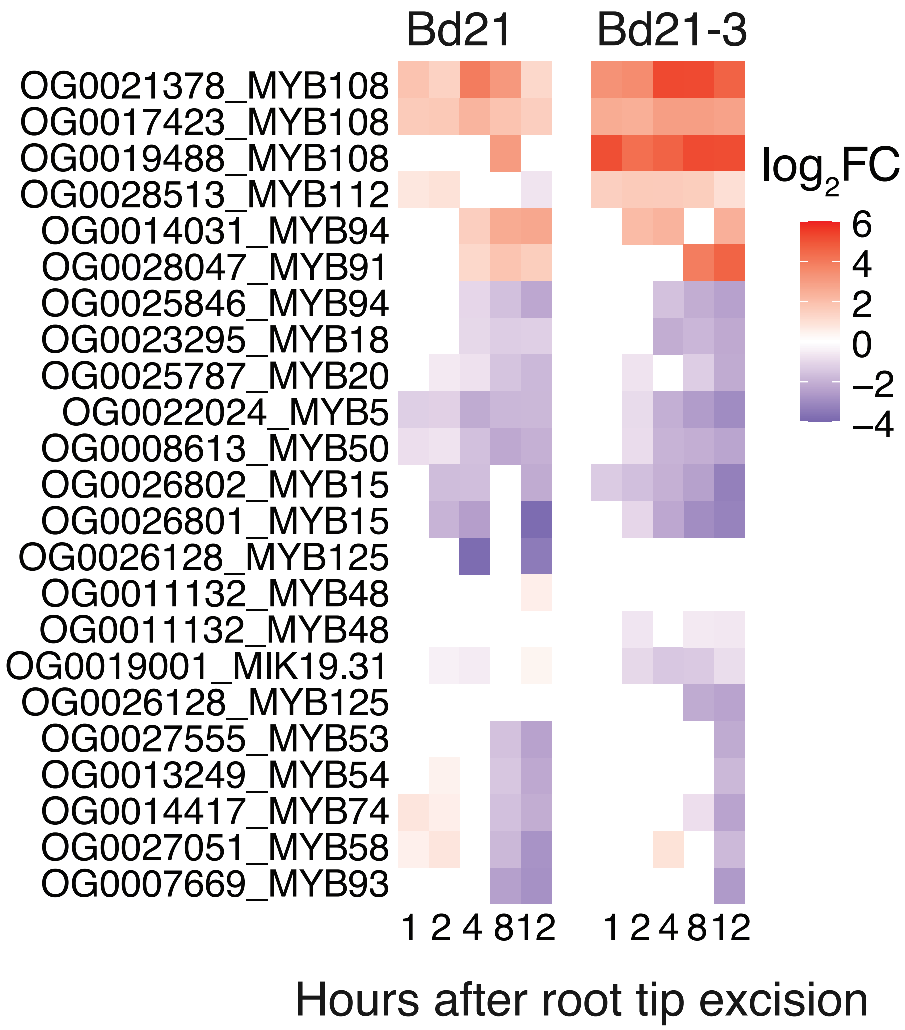

Figure S13. Expression dynamics of selected MYB transcription factor homologs following root tip excision. The heatmaps display the $\log_2$ fold-change at 1, 2, 4, 8, and 12 hours for Bd21 and Bd21-3. MYB gene names are given together with the orthogroup identifier. Corresponding gene identifiers are available in Table S6.

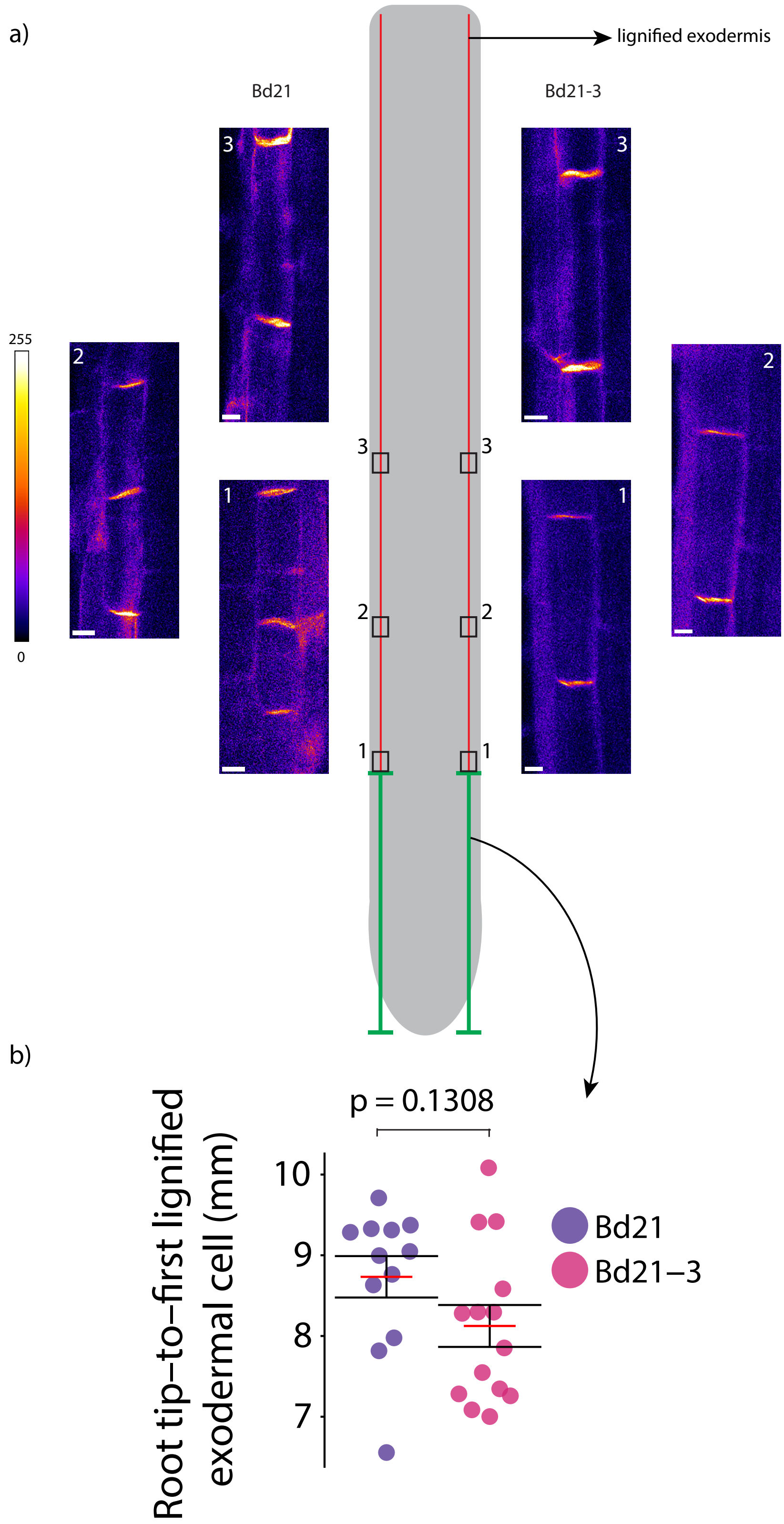

Figure S14. Bd21 and Bd21-3 show similar exodermis lignification patterns along the root tip–base axis. (a) Representative confocal images of whole mounted roots stained with BF (lignin) from 6 DAG *Brachypodium distachyon* accessions Bd21

and Bd21-3. Roots with similar length were positioned in parallel for consistency. Lignification patterns in the exodermis were assessed at increasing distances from the root tip and appear comparable between accessions across different developmental zones. A total of 12 seedlings per accession were analyzed across two biological replicates (≥6 seedlings per replicate). Scale bars: 10 μm. (B) Quantification of the distance from the root tip to the first fully lignified common anticlinal cell wall between adjacent exodermal cells. P-value is given for the result of a Wilcoxon rank sum test.

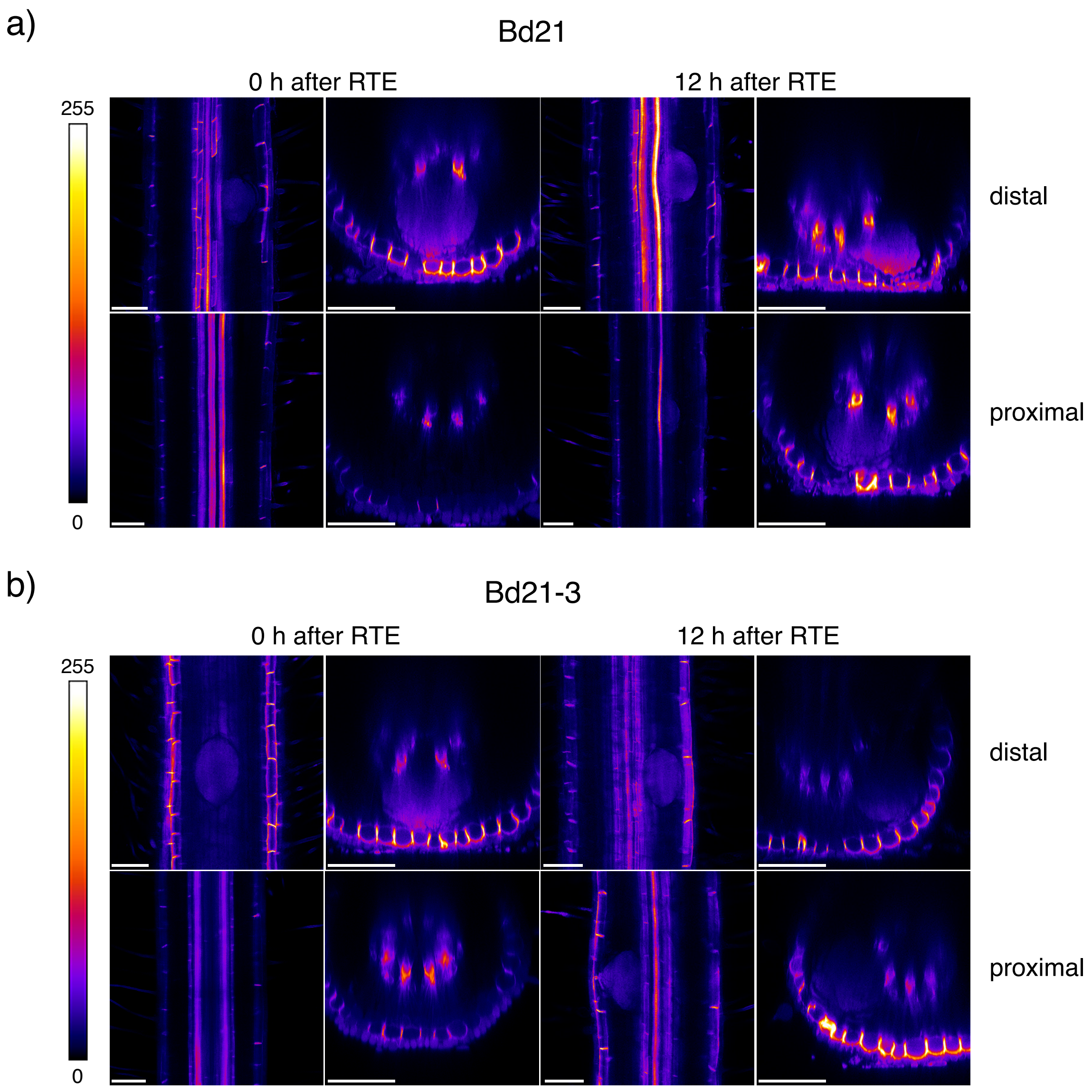

Figure S15. Accession-specific patterns of exodermis lignification during lateral root emergence. (a, b) Representative confocal Z- and Y-axis images of whole-mounted roots stained with basic fuchsin (BF) for lignin visualisation, collected at 0 h, 12 h, and 40 h after RTE. Images correspond to *Brachypodium distachyon* accessions (A) Bd21 and (B) Bd21-3. Roots were obtained from 6 DAG seedlings and mounted with similar orientation and length for consistency. A total of 20 seedlings per

accession and time point were analyzed across two biological replicates (≥10 seedlings per replicate), scale bars: 100 μm.

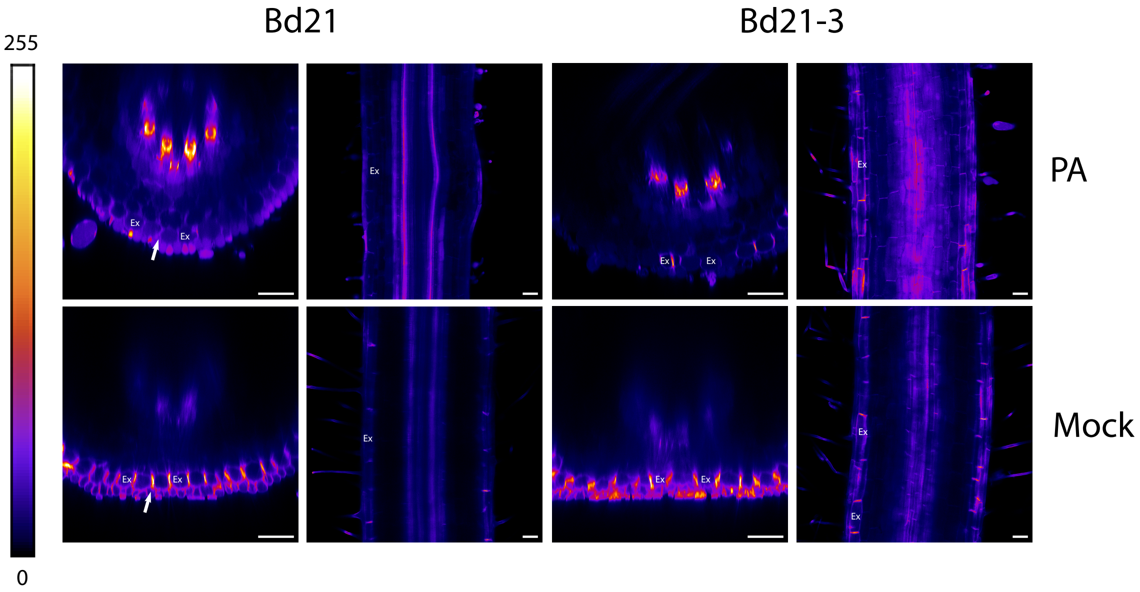

Figure S16. Reduced exodermal lignin accumulation in Brachypodium roots following piperonylic acid (PA) treatment. Representative confocal Z- and Y-axis images of whole-mounted roots stained with basic fuchsin (BF) to visualize lignin in two Brachypodium distachyon accessions, Bd21 and Bd21-3 after PA exposure. Roots grown in the presence of PA for 60 h displayed markedly reduced lignin deposition in the exodermis (Ex) compared with mock-treated controls. Arrowheads indicate the common cell wall between adjacent exodermal cells, where lignin deposition differs between treatments. Roots were mounted with similar orientation and length for consistency. Images were acquired from the same developmental zone, approximately 1.5 cm from the root tip. A total of 3 seedlings per accession and treatment were analysed. Scale bars: 50 μm.

References

Coomey JH, Mackinnon KJM, Mccahill IW, Khahani B, Handakumbura PP, Trabucco GM, Mazzola J, Leblanc NA, Kheam R, Hernandez-Romero M, *et al*. 2024. Mechanically induced localisation of SECONDARY WALL INTERACTING bZIP is associated with thigmomorphogenic and secondary cell wall gene expression. *Quantitative plant biology* 5.
